# Feature Selection Based on Information Entropy for Accurate Detection of Optical Fiber End-Face Defects

**DOI:** 10.3390/e28040462

**Published:** 2026-04-17

**Authors:** Longbing Yang, Quan Xu, Min Liao, Kang Sun, Rujie Xiang, Haonan Xu

**Affiliations:** 1 School of Mechanical Engineering, Xihua University, Chengdu 610039, China; lonbinyang@163.com (L.Y.); sunkang@stu.xhu.edu.cn (K.S.); m18982303637@163.com (R.X.); jackkingblabla@outlook.com (H.X.); 2 Modern Agricultural Equipment Research Institute, Xihua University, Chengdu 610039, China; liaomin@mail.xhu.edu.cn

**Keywords:** information entropy, multimode fiber, end-face defect detection, machine vision

## Abstract

Multimode fibers with core diameters of 50 μm and 62.5 μm are the core media for short-distance, low-cost, and high-bandwidth optical transmission scenarios. Currently, the detection of their end-face defects is still mainly based on manual microscopic inspection. Most of the existing machine vision detection schemes are aimed at polarization-maintaining fibers (POL), which are easily interfered with by impurities and have insufficient accuracy and efficiency. This study introduces the information entropy in information theory as a constraint for feature selection, proposes the WGMOS digital image detection method, and optimizes the entire process of image acquisition, correction, filtering, adaptive segmentation, and feature extraction. By minimizing the information entropy of background noise and maximizing the information content of defect features, interference is suppressed. Experiments show that compared with the POL detection method, this scheme can exclude more impurities, with the image equalization value increased by ≥38.20% and the signal-to-noise ratio increased by ≥6.0%. It can achieve efficient and accurate detection of multimode fiber end-face defects.

## 1. Introduction

**Figure 1:**
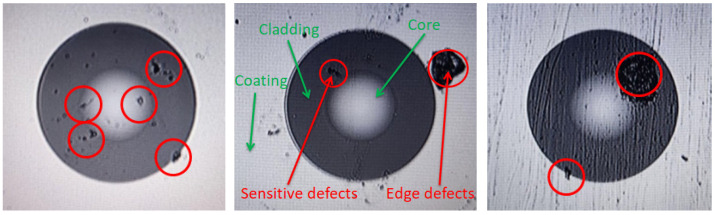
Example of multimode fiber end-face defects.

Optical fibers are thin fibers that transmit optical signals using the principle of total internal reflection of light, usually made of glass or plastic. Since the 1970s, optical fiber communication technology has developed rapidly and has now become one of the core technologies of modern communication networks. It has significant advantages such as high-speed data transmission, long-distance transmission, and strong anti-interference ability, and is widely used in the communication field [1,2]. Defects on the end-faces of multimode optical fibers usually cause problems such as increased insertion loss, decreased return loss, deteriorated communication performance, and even permanent damage to devices. Currently, most communication module manufacturers mainly use high-magnification electron microscopes for manual visual inspection of optical fiber end faces. This method has deficiencies in terms of accuracy and efficiency, and the labor cost is high [3]. The breakthrough development of machine vision and digital image processing technology [4,5] provides more possibilities for improving the detection efficiency and robustness of multimode optical fiber end-face detection [6]. In the actual production and manufacturing process, the defects on the end faces of multimode optical fibers mainly manifest as water droplets, oil stains, impurities, scratches, etc. Their shapes and sizes vary greatly, and there is also the problem of uneven background (such as Figure 1) [7]. As shown in Figure 1, all three images display obvious defects. As illustrated in the middle image, the fiber end face consists of the core, cladding, and coating. Defects in the core and its adjacent regions are considered Sensitive defects. Defects in the cladding and its adjacent regions have a lesser impact on optical signal transmission compared to core defects, and this impact decreases as the radius increases. Defects at the interface between the cladding and the coating have a minor effect and require detection, while defects over a larger area have almost no effect and do not require detection. In other words, defects outside the cladding (excluding the interface between the cladding and the coating) can be detected but do not require detection. This paper combines information entropy in information theory as a feature selection constraint and proposes a machine vision method for accurate detection of end-face defects of multimode optical fiber connectors—the WGMOS detection method. This method aims to maximize the defect detection rate in the multimode optical fiber end-face detection scenario [8,9]. The WGMOS detection method is a computer image processing technology based on morphology. Its core optimization goals are to minimize the information entropy of background noise and maximize the information volume of defect features, eliminating interference factors and accurately extracting defect features. This is specifically achieved through the following steps: First, convert the originally captured image into a grayscale image; second, correct it and complete noise filtering; third, use an adaptive threshold to complete image segmentation; finally, extract edge-based features. By comparing the grayscale value differences in the regions around each pixel, it ensures the elimination of interference factors and the successful extraction of defect features [10,11]. The primary defects identified in this inspection plan include non-removable foreign objects or stains and scratches.

Basic Definitions and Calculations of Information Entropy: Information entropy is a core metric in Shannon’s information theory that quantifies the uncertainty of information. For an 8-bit grayscale digital image, the formula for calculating grayscale information entropy is: $H=-\sum_{i=0}^{255}\;p(i)\cdot {log}_{2}p(i)$. Here, $p\left(i\right)$ represents the proportion of pixels with grayscale value i out of the total number of pixels, expressed in bits. A higher entropy value indicates a more dispersed grayscale distribution, greater textural complexity, and higher information content; conversely, a lower entropy value indicates a more concentrated grayscale distribution.

### The Application of Information Entropy

Adaptive Segmentation Stage: The total entropy of the segmented foreground (defective regions) and background (normal regions) is used as a performance metric. A higher total entropy indicates better discrimination between the two types of regions, allowing the optimal segmentation threshold to be determined and avoiding issues such as over-segmentation and under-segmentation.

Feature Extraction Stage: On one hand, information entropy is used as a constraint for feature selection, retaining only highly discriminative features with entropy values within a reasonable range while eliminating ineffective features that lack discriminative power or are excessively redundant, thereby reducing computational load. On the other hand, the entropy value of the gray-level co-occurrence matrix under the texture feature dimension can serve directly as a core discriminative feature for distinguishing between defective and normal regions, supporting detection and classification.

The primary objective of this research proposal is to use “information entropy” as a filter to automatically identify, from a set of image features, those that best reveal scratches and speckles. These images are then fed into a lightweight classifier, enabling the machine to standardize and accelerate the process that relies on human visual expertise. Its significance lies in the fact that it is not merely a showcase of algorithms, but rather a tool that helps factories intercept “invisible surface defects” before shipment—in milliseconds, at low cost, and in a manner that is fully explainable.

The main contributions of this paper are:

(1) A WGMOS inspection method for multimode fiber end-face defect detection based on machine vision is proposed, and the specific implementation method, experimental process, and experimental equipment of the WGMOS inspection method are described and illustrated in detail.
(2) Combining machine vision and digital image processing techniques improves the equalization value and signal-to-noise ratio of multimode fiber end-face defect detection and eliminates the influence of more interfering factors. The experimental results show that the equalization value of the WGMOS detection method for regional defects is improved by 38.20% and the signal-to-noise ratio is improved by 6.0% compared with the POL detection method [12]. Meanwhile, the WGMOS detection method excludes more interfering impurities than the POL detection method, and the detected features are clearer.
(3) The remainder of this paper is structured as follows. Section 2 provides a detailed exposition of the principles and implementation of the WGMOS detection method. In Section 3, the experimental process and quality assessment criteria for multimode fiber end-face defect detection are described in detail. In Section 4, the paper presents the detection of end-face defects of multimode optical fibers by the WGMOS detection method and gives quantitative results in comparison with the existing techniques. Section 5 summarizes the discussion of the paper.

## 2. Materials and Methods

Modern image processing technology achieves preset goals by analyzing, processing, and transforming images, and is widely applied in fields such as computer vision and industrial automation. In response to the demand for precise detection of optical fiber end-face defects, this research introduces information entropy from information theory as the core criterion for feature screening. In the image processing chain, defect features with high information content are preferentially retained, and redundant background noise with low entropy values is filtered. Feature extraction is a core step, which can support accurate identification and classification of defects. After obtaining the original image, pre-processing needs to be completed first to eliminate redundant information and enhance key defect information. The pre-processing chain adopted in this research is: grayscale conversion–gamma correction–image filtering–adaptive threshold segmentation–edge extraction [13].

### 2.1. Grayscale Conversion

**Figure 2:**
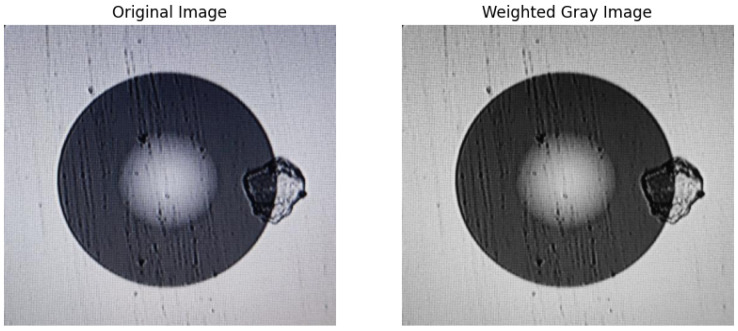
Before and after grayscaling of multimode fiber end-face images.

Grayscale conversion can transform a color image into a single-channel grayscale image. While simplifying calculations, it ensures the statistical consistency of pixel information content, providing a unified numerical basis for subsequent feature screening based on information entropy. Commonly used grayscale conversion methods include the maximum value method, the average value method, and the weighted average method. Among them, the weighted average method takes into account the human eye’s sensitivity to colors and can obtain a processing result that is more in line with visual perception [14]. As a pre-processing step, it can lay the foundation for subsequent operations such as image segmentation, identification, and analysis. The weighted average method assigns different weights to the three RGB components according to the importance of visual perception for weighted operations. Since the human eye is most sensitive to green and least sensitive to blue, by performing a weighted average on the three RGB components according to the following formula, a grayscale image with a more reasonable grayscale value distribution and a higher discrimination degree between defect and background information entropy can be obtained [15]. The formula is as follows:

(1) $$Gray\left(i,j\right)=0.299\times R\left(i,j\right)+0.578\times G\left(i,j\right)+0.114\times B\left(i,j\right)$$

where R(x, y) is the value of the pixel’s red channel, G(x, y) is the value of the pixel’s green channel, and B(x, y) is the value of the pixel’s blue channel. Since the conversion logic of the weighted average method for color images is more suitable for the entropy-value feature screening requirements of this research and the resulting grayscale image has the best effect, the weighted average method is selected in this technology to perform grayscale processing on the original optical fiber end-face image [16]. The grayscale conversion of the original image is achieved by programming in image processing software. The original image and the grayscale image processed by the weighted average method are shown in Figure 2.

### 2.2. Gamma Correction

**Figure 3:**
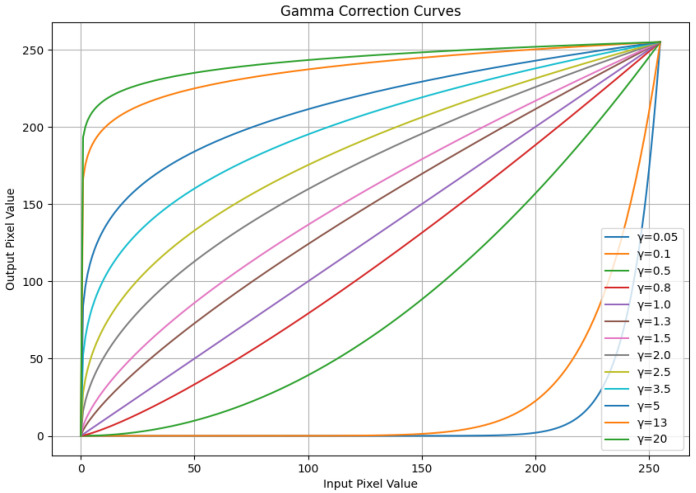
Calibration curves for different gamma values.

**Figure 4:**
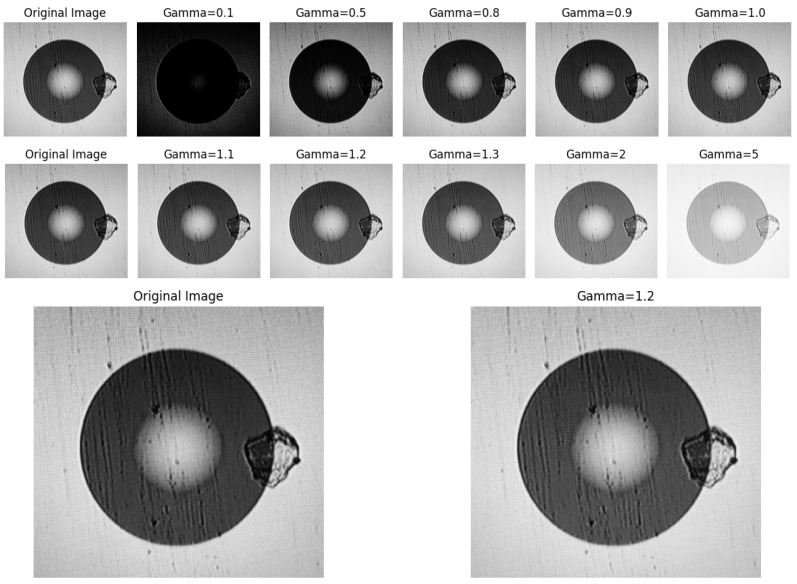
Comparison of multimode fiber end-face images with different gamma values.

The grayscale multimode fiber end-face images generally have the problem of uneven light and dark distribution, which is likely to lead to insufficient distinction in information entropy between background noise and defect features, affecting the accuracy of subsequent feature selection based on information entropy. Therefore, in this step, gamma correction is introduced to optimize the pixel brightness distribution. Its mathematical expression is $O=I^{\gamma }$, where *O* is the output pixel value, I is the input pixel value, and γ is the gamma value, usually taking values between 0.5 and 2.5 [17]. When γ is less than 1, the low-brightness area of the image is stretched, enhancing the dark details; when γ is equal to 1, the image brightness remains unchanged; when γ is greater than 1, the high-brightness area of the image is stretched, enhancing the bright details. The correction curve of gamma correction is shown in Figure 3, and the multimode fiber end-face images under different gamma correction values are shown in Figure 4. From the perspective of the difference in information entropy distribution, when the gamma value is 1.2, the entropy difference between the defect area and the clean background area of the processed image is the largest. It can not only display more defect detail features but also eliminate the influence of some low-information interference factors [18], adapting to the feature selection requirements based on information entropy in this study. Therefore, a gamma value of 1.2 will be adopted for the correction of the grayscale image in this technology.

### 2.3. Image Filtering

**Figure 5:**
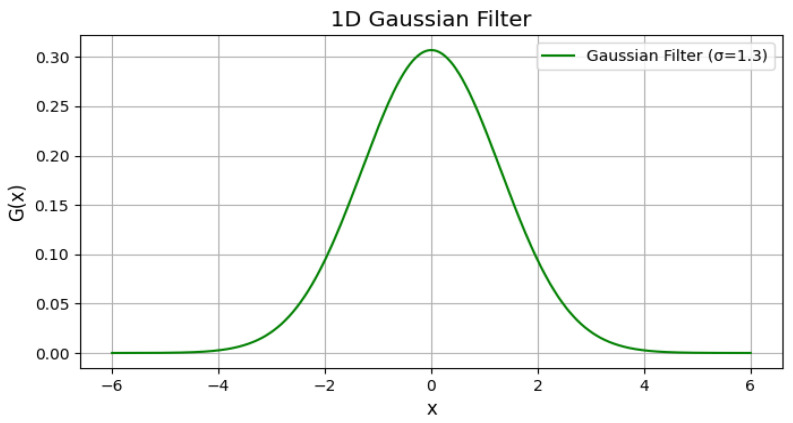
One-dimensional Gaussian function curve when σ = 1.3.

After completing the gamma correction pre-processing step, the resulting image still contains random noise, which can easily interfere with the statistical accuracy of the information entropy in local regions. This leads to errors in subsequent defect feature extraction and matching based on information entropy, thus affecting the accuracy of system identification and positioning [19]. In the field of image processing, spatial filtering is commonly used to eliminate or reduce noise, filter out redundant interference with low entropy values, and improve the signal-to-noise ratio of effective defect features, achieving the effects of suppressing interference and improving image quality [20]. Among linear filters, Gaussian filtering is the most suitable for the entropy value screening requirements of this study in terms of suppressing normally distributed noise. Its core principle is as follows: Gaussian filtering is a linear smoothing filter. A neighborhood of an appropriate size is selected centered on the target pixel, and the pixels within the neighborhood are weighted according to the Gaussian distribution, which can effectively eliminate Gaussian noise that follows a normal distribution. The mathematical expression of the one-dimensional Gaussian function is:

(2) $$G\left(x\right)=\frac{1}{\sqrt{2\pi }\sigma }\exp\left(-\frac{x^{2}}{2\sigma^{2}}\right)$$

where σ is the variance, which plays a crucial role in the filter width and smoothness of Gaussian filtering. The filtering effect varies with different values of σ [21]. The smaller the σ, the more concentrated the density distribution, the more concentrated the weighted calculation around the center of the neighborhood, the smaller the effective filtering range, and the weaker the ability to suppress noise. Conversely, the more dispersed the density distribution, the larger the effective filtering range, the stronger the ability to suppress noise, and the more efficient the screening of low-entropy random noise [22]. The image of the one-dimensional Gaussian function when σ = 1.3 is shown in Figure 5. The two-dimensional Gaussian function is often used for filtering digital images. The expression of the two-dimensional Gaussian function is as follows:

(3) $$G\left(x,y\right)=\frac{1}{2\pi \sigma^{2}}\exp\left(-\frac{x^{2}+y^{2}}{2\sigma^{2}}\right)$$

**Figure 6:**
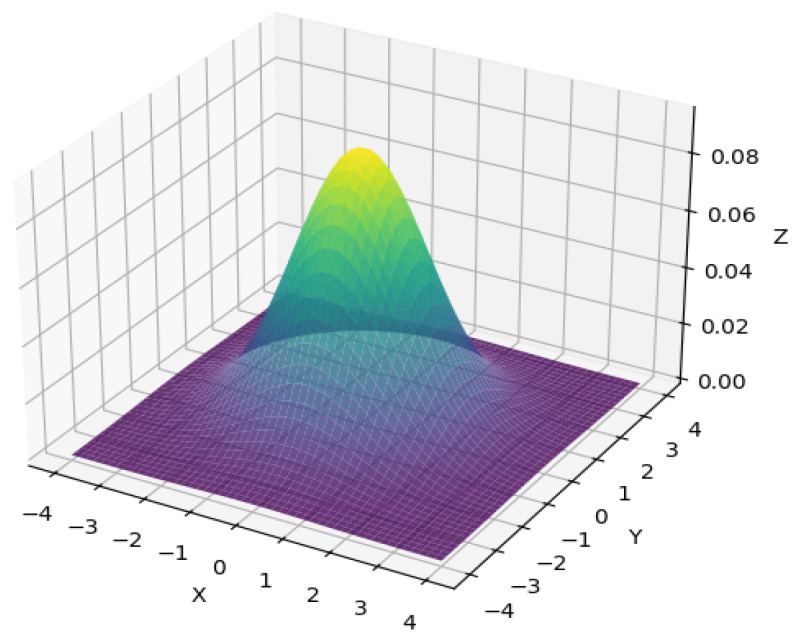
2D Gaussian function in 3D plane curve when σ = 1.3.

Two-dimensional Gaussian filtering has the same filtering effect in any direction on the image. By utilizing the separable property of the Gaussian function in multiple directions, the image can be convolved with the one-dimensional Gaussian function in the vertical and horizontal directions, reducing the computational complexity. The image of the two-dimensional Gaussian filtering function in three-dimensional space coordinates when σ = 1.3 is shown in Figure 6.

In addition to Gaussian filtering, commonly used linear filtering schemes also include mean filtering and median filtering. To select the filtering method that best suits the information entropy-based feature selection requirements of this study, a comparative analysis of the principles and processing effects of these three types of filtering is carried out: The mean filtering method [23] mainly uses a filtering model to perform a convolution operation on the original image and replaces the pixel values in the original image according to the following principle formula:

(4) $$g\left(x,y\right)=\frac{\sum f\left(x,y\right)}{m}$$

where f(x, y) is the pixel value of the original image, g(x, y) is the pixel value calculated by the template, and m is the total number of pixels including the current pixel in the template [24]. This method easily smoothes out the local pixel gray-level differences, dilutes the high-information entropy value at the edge of the defect, and is prone to causing feature loss. Median filtering mainly uses the neighborhood template to calculate the median of the pixel values in the original image and replaces the original value with this median, which avoids image blurring and protects the high-entropy feature information at the edges [25]. The output of a two-dimensional median filter is:

(5) $$g\left(x,y\right)=med\left\{\left(x-k,x-l\right)\right\}$$

where x is the target pixel point, k is the width of the filtering template, and l is the height of the filtering template. In general, k and l take the same value and are odd numbers, and g(x, y) is the filtered image. The shape of the template neighborhood can be adjusted according to the application scenario, and common shapes include circular, elliptical, and cross-shaped.

**Figure 7:**
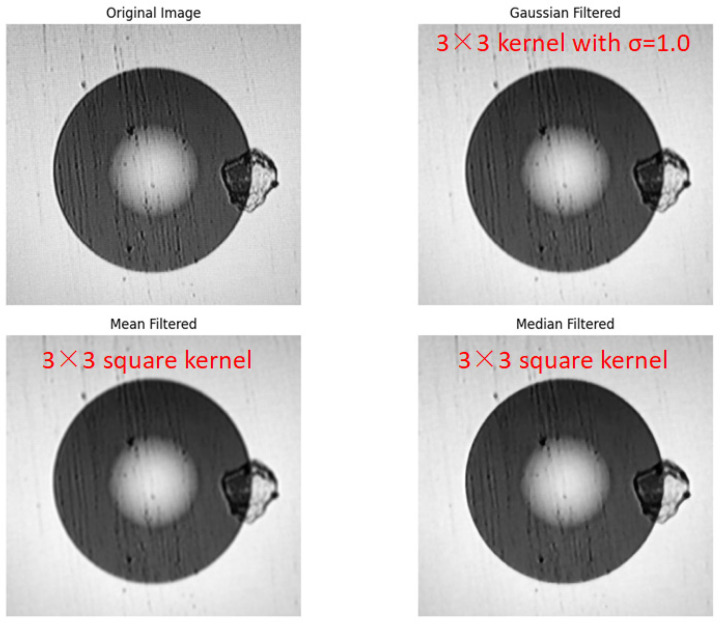
Comparison of multimode fiber end-face images under different filtering methods.

This paper combines the information entropy feature screening logic (prioritize retaining high-entropy features of defects and filtering out low-entropy interference from noise) to compare these three methods and carry out filtering experiments. As shown in Figure 7, it can be seen that the median filtering effect is better. On the one hand, the image is not significantly blurred, and the high-entropy features at the edges of the defects are completely retained. On the other hand, it can effectively filter out the interference of low-entropy salt-and-pepper noise. Gaussian filtering and mean filtering cannot eliminate salt-and-pepper noise, and their denoising effects are not as good as that of median filtering. Moreover, they blur the image and lose high-information image detail data, which is not conducive to the later extraction of defect features based on information entropy [26]. Therefore, this paper adopts median filtering to filter and denoise the image.

### 2.4. Otsu Threshold Segmentation

After completing median filtering for noise reduction, it is necessary to separate the defect features (regions with high information content and high information entropy) in the end-face image from the clean end-face background (regions with low entropy and low redundancy) through threshold segmentation, providing a classification basis for subsequent feature selection based on information entropy. In this step, the Otsu threshold segmentation method is selected, which is suitable for this requirement. This method is particularly useful when there is a bimodal histogram between the image background and the foreground. Its basic idea is to select an optimal threshold to segment the image, and this threshold can maximize the inter-class variance. Essentially, it is equivalent to maximizing the inter-class information entropy difference between the foreground defects and the background region, which can fully ensure the distinguishability between the two types of regions. This algorithm is considered the most effective threshold selection algorithm in image segmentation because it is simple and independent of image brightness and contrast. Therefore, it is a widely used method in digital image processing [27].

In a two-dimensional histogram, if $B_{0}$ represents the target (defect high-entropy region) and $B_{1}$ represents the background (low-entropy clean region), they have two different probability density distributions. Assuming the threshold is (x, y), the probabilities of their occurrence are respectively 0 and 1. The Otsu model uses the same framework as the previous research of Yang [12]:

(6) $$\omega_{0}=P_{r}\left(B_{0}\right)=\sum_{i=1}^{x}\;\sum_{j=1}^{y}\;p_{ij}=\omega_{0}\left(x,y\right)$$

(7) $$\omega_{1}=P_{r}\left(B_{1}\right)=\sum_{i=x+1}^{L}\;\sum_{j=y+1}^{L}\;p_{ij}=\omega_{1}\left(x,y\right)$$

Their corresponding mean vectors are:

(8) $$\mu_{0}={\left(\mu_{0i},\mu_{0j}\right)}^{T}={\left(\sum_{i=1}^{x}ip_{r}\left(i/B_{0}\right),\sum_{j=1}^{y}ip_{r}\left(j/B_{0}\right)\right)}^{T}={\left(\frac{\mu_{i}\left(x,y\right)}{\omega_{0}\left(x,y\right)},\frac{\mu_{j}\left(x,y\right)}{\omega_{0}\left(x,y\right)}\right)}^{T}$$

(9) $$\mu_{1}={\left(\mu_{1i},\mu_{1j}\right)}^{T}={\left(\sum_{i=y+1}^{L}ip_{r}\left(i/B_{1}\right)\sum_{j=y+1}^{L}ip_{r}\left(j/B_{1}\right)\right)}^{T}$$

The total mean vector on the 2D histogram is:

(10) $$\mu_{T}={\left(\mu_{Ti},\mu_{Tj}\right)}^{T}=\sum_{i=1}^{L}\sum_{j=1}^{L}\left(i\times p_{ij}\right)$$

When the probability of movement away from the diagonal of the histogram is negligible, the histogram is displayed, and it can be demonstrated that L has $p_{ij}\approx 0$. Consequently, it can be deduced that L has $p_{ij}\approx 0$.

When the probability of deviation from the histogram diagonal is negligible, L has $p_{ij}\approx 0$, then it is easy to show that:

(11) $$\omega_{0}+\omega_{1}\approx 1,\mu_{T}\approx \omega_{0}\mu_{0}+\omega_{1}\mu_{1}$$

Define a discretization matrix between classes:

(12) $$S_{B}=\sum_{k=0}^{1}p_{r}\left(C_{k}\right)\left[\left(\mu_{k}-\mu_{T}\right){\left(\mu_{k}-\mu_{T}\right)}^{T}\right]$$

In the context of employing the trace of $S_{B}$ As a discretization measure between classes, the following holds:

(13) $${t_{r}S}_{B}=\omega_{0}\left[{\left(\mu_{0i},\mu_{Ti}\right)}^{2}+{\left(\mu_{0j},\mu_{Tj}\right)}^{2}\right]+\omega_{1}\left[{\left(\mu_{1i},\mu_{Ti}\right)}^{2}+{\left(\mu_{1j},\mu_{Tj}\right)}^{2}\right]$$

Combining Equation (8) with Equation (10) gives:

(14) $${t_{r}S}_{B}=\frac{{\left[{\mu_{Ti}\omega }_{0}\left(x,y\right)-\mu_{i}\left(x,y\right)\right]}^{2}+{\left[{\mu_{Tj}\omega }_{0}\left(x,y\right)-\mu_{j}\left(x,y\right)\right]}^{2}}{\omega_{0}\left(x,y\right)\left[1-\omega_{0}\left(x,y\right)\right]}$$

Similar to one-dimensional Otsu, the optimal threshold $\left(x^{\prime },y^{\prime }\right)$ satisfies the following equation:

(15) $${t_{r}S}_{B}\left(x^{\prime },y^{\prime }\right)=\underset{1\leq s,t<L}{\max}\left\{{t_{r}S}_{B}\left(x,y\right)\right\}$$

**Figure 8:**
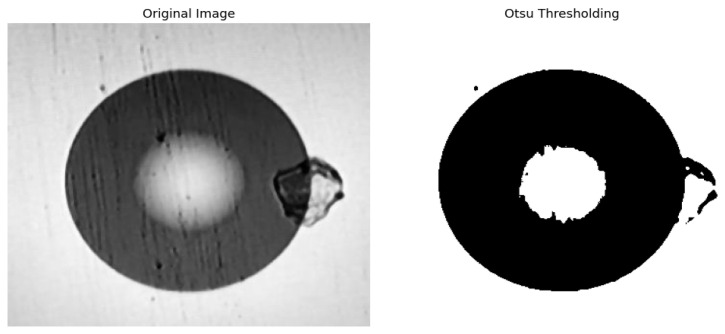
Comparison of images before and after Otsu threshold segmentation.

The Otsu threshold segmentation process is predicated on the concept of grouping. Initially, the number of gray levels in the image is divided into two parts based on the gray levels. This is done so that the difference between the gray values in the two parts is maximized, and the difference between the gray values in each part is minimized. Subsequently, the variance is calculated, and an appropriate gray level is identified for segmentation [28]. Otsu thresholding segmentation divides the image into two parts, background and foreground, based on the gray-level characteristics of the image. An image of a multimode fiber end-face after Otsu thresholding segmentation is shown in Figure 8.

### 2.5. Edge Detection

The purpose of edge detection of an image is to identify regions of an image where the pixel intensity varies significantly, including the contours of the region, texture variations, or boundaries between different objects. Edge detection plays a key role in applications such as image segmentation, target recognition, and feature extraction [29]. There are the following common methods for image edge detection.

The Scharr algorithm is an image edge detection operator that convolves the environment with the image to detect the edges in the image. The Scharr algorithm has better rotation invariance and smaller edge response error compared to the Sobel algorithm [30]. The Laplace operator is an edge detection operator based on second-order derivatives, which detects edges by calculating the second-order derivatives of image pixels. The Laplace operator is sensitive to edges and can reveal details and rapidly changing regional features [31]. The 2D image function of the Laplace operator is the second-order isotropic derivative, which has the following definition:

(16) $$\nabla^{2}f\left(x,y\right)=\frac{\partial^{2}f}{\partial x^{2}}+\frac{\partial^{2}f}{\partial y^{2}}$$

In images, the equations are represented in discrete form:
The one-dimensional case:

(17) $$\frac{\partial^{2}f}{\partial x^{2}}=f\left(x+1\right)-2\times f\left(x\right)+f\left(x-1\right)$$

(18) $$\frac{\partial^{2}f}{\partial y^{2}}=f\left(y+1\right)-2\times f\left(y\right)+f\left(y-1\right)$$

Two-dimensional situation:

(19) $$\nabla^{2}f\left(x,y\right)=\left[f\left(x+1,y\right)+f\left(x-1,y\right)+f\left(x,y+1\right)+f\left(x,y-1\right)\right]-4f\left(x,y\right)$$

The Laplacian operator, being a differential operator, has been demonstrated to enhance areas of an image that show rapid changes in grayness and attenuate areas that show slow changes in grayness. The enhancement of the image can be achieved by selecting the Laplacian operator to process the original image, thereby creating an image that accounts for rapid changes in grayness. The Laplacian image is then superimposed on the original image to create an enhanced image [32].

Edge detection is based on the Canny operator. Canny edge detection is a multi-step edge detection algorithm that can better suppress noise and detect clear edges [33]. The core of the Canny algorithm, however, uses a Gaussian filter to smooth the image, determines the amount and direction of the image’s gradient, applies non-maximum suppression, and uses a double threshold to determine edge features.

**Figure 9:**
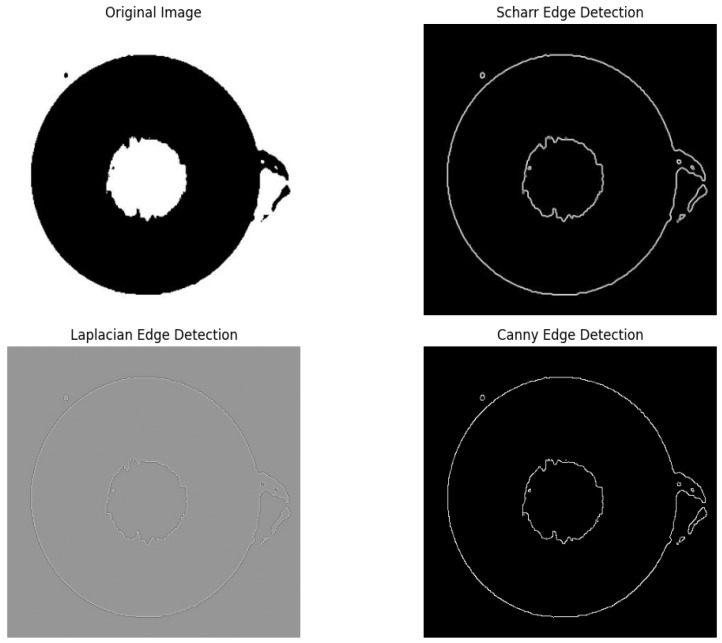
Comparison of multimode fiber end-face images under different edge detection methods.

The comparison results of the above three methods are shown in Figure 9. It can be seen from the images that the Scharr algorithm gives the best results with clear edge information and is compared with Canny edge detection, which has the problem of breakpoints. The edge detection of the Laplacian operator blurs the defective points and does not extract the defective information. Thus, in this paper, the Scharr operator is used for edge detection of images.

## 3. Materials and Experiments

This section commences with a presentation of the equipment and software employed in the experimental process. This is followed by a comprehensive account of the defect detection zones and detection criteria on the front face of the multimode fiber.

### 3.1. Experimental Data Set

This experimental study used the following equipment and software:

- Camera: Basler a2A1920-51gcBAS GigE (Basler, Ahrensburg, Germany);
- Camera lens: Moritex MML05-HR110D (Moritex, Yokohama, Japan);
- Image capture card: LR-Link LRES2004PT-POE (Shenzhen LR-Link Electronics Co., Ltd., Shenzhen, China);
- Point source: Trustauto CP2-8-3-WGJX (Trust Auto, Guangzhou, China);
- Ring Light Source: Trustauto CR-6090-WHSYS (Trust Auto, Guangzhou, China);
- Point light controller: Trustauto SD2-24W64-4T-5V4I (Trust Auto, Guangzhou, China);
- Ring Light Controller: Trustauto SD2-24W64-4TGJX (Trust Auto, Guangzhou, China);
- End-face Magnifier: DIMENSION Easycheck Integreated Inspector, model: EC200KC INPUT: DC12 V 3.0 A S/N: ECCAH3900 (Dimension, New Bedford, MA, USA);
- Fiber Optic Connector: multimode fiber2.5/PC (Ningbo Lianke Communication Equipment Co., Ltd., located in Ningbo, Zhejiang);
- Monitor: AOC LCD monitor, model: 24B1XH (Fujian Jielian Electronics Co., Ltd., Fuzhou, China).

The experiment was conducted on a designated host computer Win10 i5-5200U CPU@ 2.20 GHz 64 bit. The proposed WGMOS model was programmed in Python 3.11 on the PyCharm Community Edition 2023.1.6 OpenCV-python-4.11.0.86 platform.

**Figure 10:**
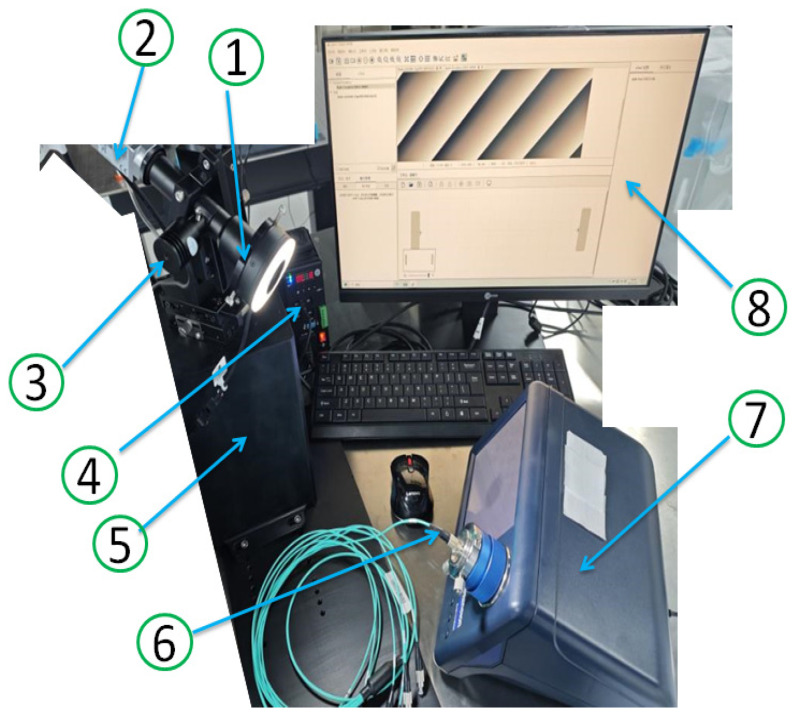
Experimental platform for multimode fiber end-face inspection. (1) Ring light source; (2) Camera; (3) Point source; (4) Ring light controller; (5) Structural support components; (6) Multimode fiber; (7) End-face magnifier; (8) Monitor.

Figure 10 shows the experimental setup used in this program.

### 3.2. Detecting and Evaluating Fiber Optic End-Face Defects by Region and Standard Segmentation

**Figure 11:**
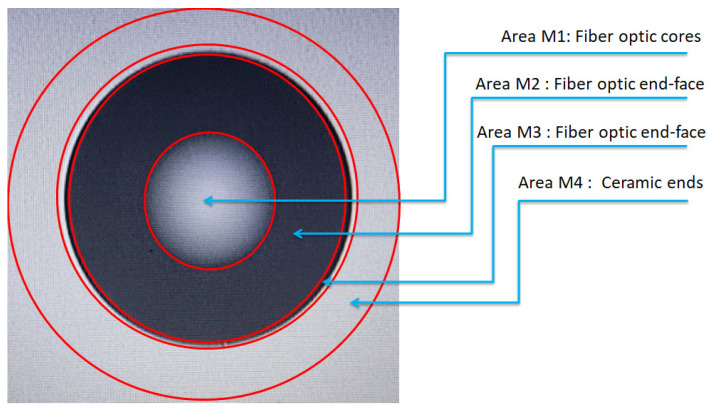
Illustration of multimode fiber end-face area division.

**Figure 12:**
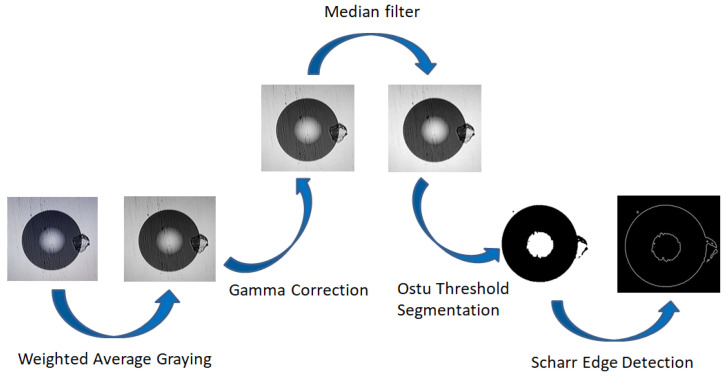
Flowchart of multimode fiber end-face image WGMOS inspection method.

In the current manufacturing process, the multimode fiber is divided into four regions for fault detection. These regions are designated as the core region M1, fiber end-face region M2, fiber end-face region M3, and ceramic end-face region M4, as illustrated in Figure 11. Each region exhibits a distinct beam-splitting pattern and different defect detection requirements. The defect detection criteria for multimode fiber end faces are enumerated in Table 1. The compliance criteria enumerated in Table 1 exceed those stipulated in the third edition of IEC 61300-3-35:2022 [34]. The conformity criteria in Table 1 are borrowed from an existing manufacturer of optical communication modules, which implements criteria higher than the conformity requirements of the standard. The implementation of the WGMOS inspection technique for the detection of end-face defects in multimode optical fibers is shown in Figure 12. The red circles serve as boundaries for the different zones of the fiber end face; the four circles divide the end face into four zones based on importance (the classification is based on the extent to which impurities in each zone affect the transmitted signal). These four inspection zones are located within the fiber core and cladding regions and do not include the jacket.

The M4 region of the ceramic end face is where the fiber cladding meets the jacket; it is part of the cladding boundary region. A fiber has only one core, commonly referred to as the light-transmitting point (i.e., M1). M2 and M3 are both end-face regions within the cladding; they are defined by different radii, as shown in Table 1. Impurities of the same size have a greater impact on fiber transmission in the M2 region than in the M3 region. Therefore, we implemented more precise regional divisions during the manufacturing process.

## 4. Results and Discussion

**Figure 13:**
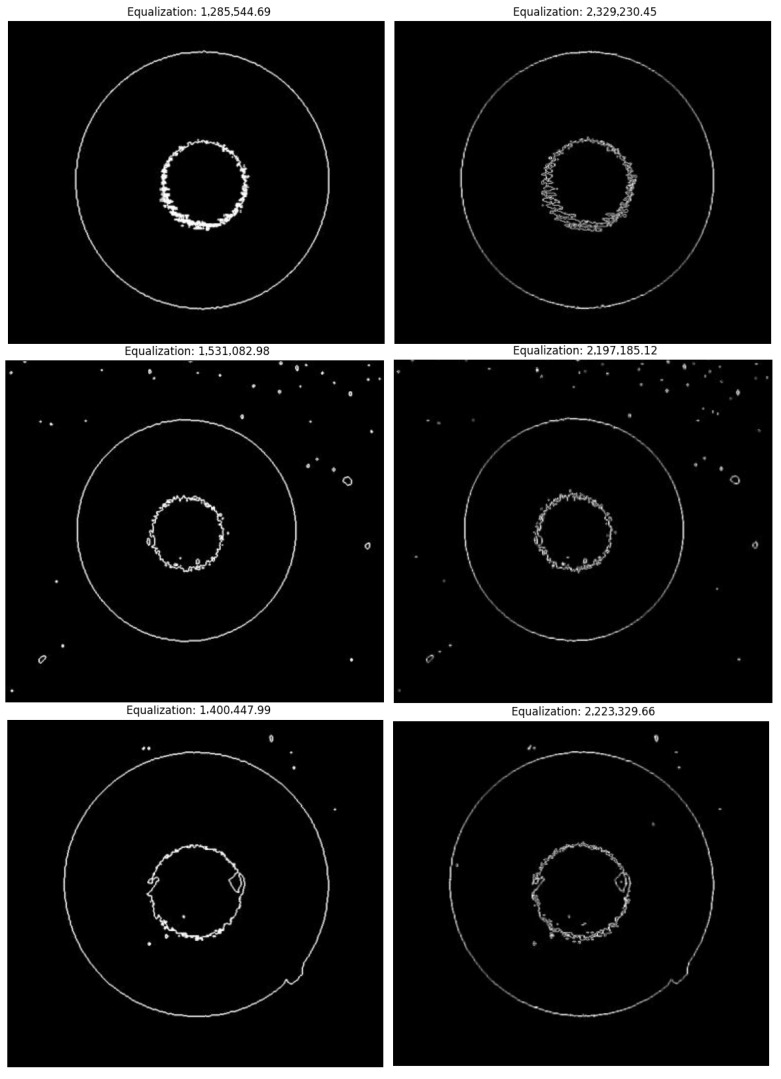

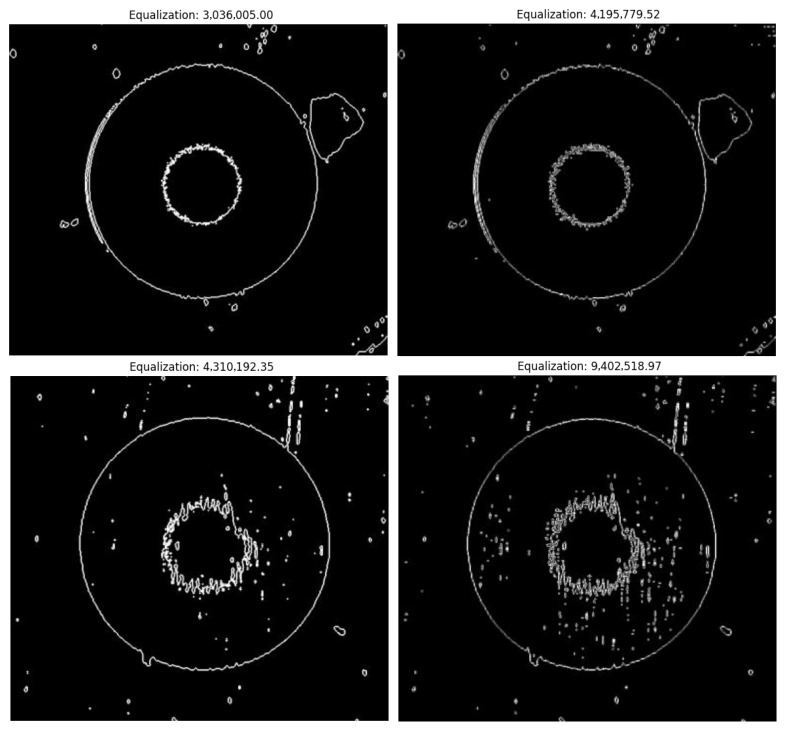
Comparison of equalization values of multimode fiber end-face images under POL detection method and WGMOS detection method.

**Figure 14:**
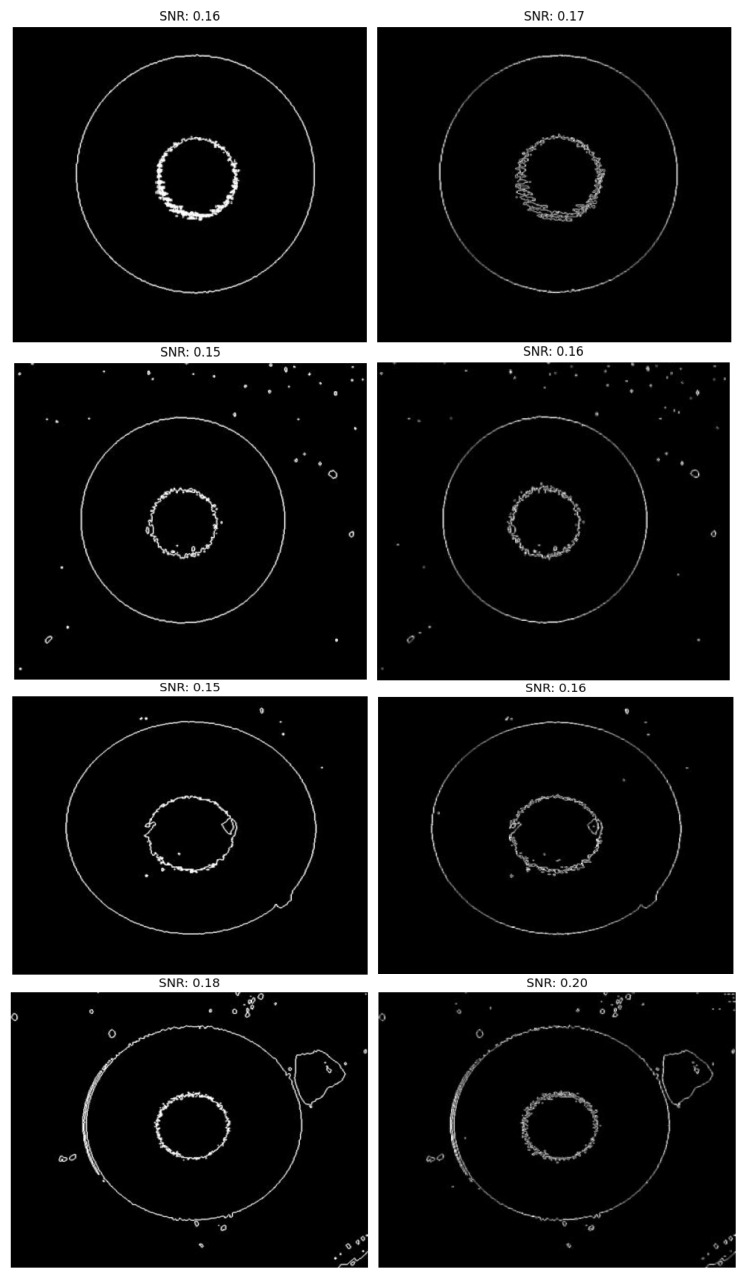

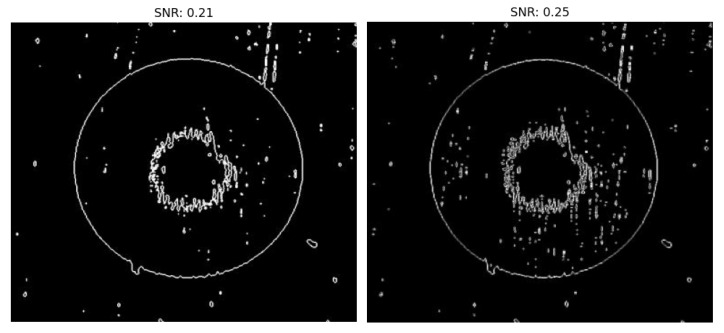
Multimode fiber end-face signal-to-noise ratio comparisons.

To verify the actual performance of the WGMOS end-face defect detection model with the introduction of information entropy feature selection constraints, a series of comparative experiments were carried out in this section. The multimode fiber samples used in this study were all collected from the production site of a manufacturing enterprise, and the test platform was designed to meet the high-entropy feature recognition requirements of multimode fiber end-face defects. Referring to the evaluation standard of multimode fiber end-face defects, a total of five groups of end-face magnified samples were collected. The number of impurities in the end-face defects of the samples was set in a gradient from less to more, covering common working conditions in actual production. To intuitively reflect the advantages of the WGMOS detection method compared with the traditional POL detection method, taking the image equalization value and signal-to-noise ratio under the information entropy constraint as the core evaluation indicators (the higher the two types of indicators, the better the discrimination between high-entropy defect features and low-entropy background and interference noise), the detection results of the two methods were compared (the left figure shows the results of the POL detection method, and the right figure shows the results of the WGMOS detection method). The specific comparison results are shown in Figure 13 and Figure 14.

**Figure 15:**
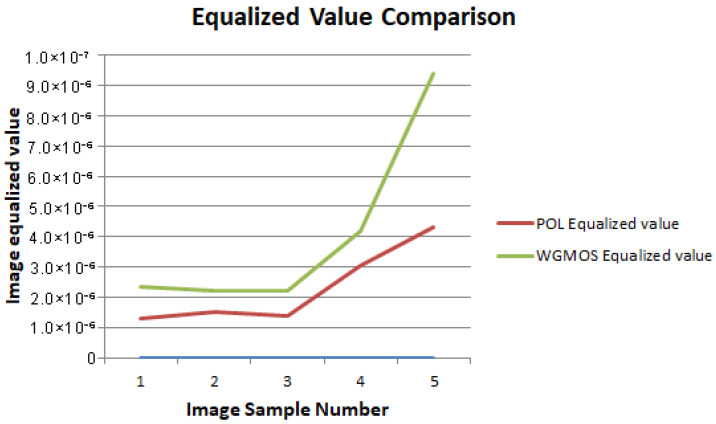
Equalized value comparison chart.

**Figure 16:**
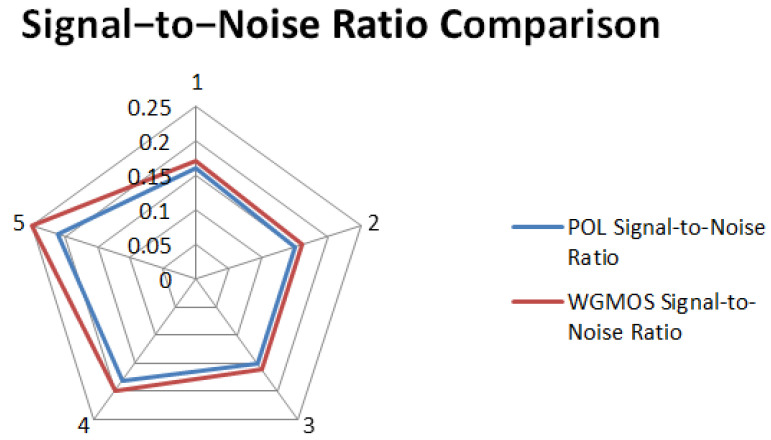
Signal-to-noise ratio comparison chart.

By calculating the image equalization values and signal-to-noise ratios of the two types of detection results, the comparative data of the defect extraction performance of the WGMOS detection method and the POL detection method were statistically obtained. The measured data of the equalization values and signal-to-noise ratios of the two methods are shown in Table 2. For an intuitive comparison of the equilibrium values and signal-to-noise ratios of the two methods, see Figure 15 and Figure 16, respectively. The experimental results show that for the WGMOS detection model optimized by information entropy feature selection, the equalization value and signal-to-noise ratio of the multimode fiber end-face detection are at least increased by 38.20% and 6.0%, respectively, compared with the POL detection method, and the two core indicators are significantly better than the traditional POL method. At the same time, when dealing with various interfering impurities on the end face of the multimode fiber, the WGMOS method can more efficiently filter out low-entropy redundant interference, and the denoising effect is significantly better than the POL method. Overall, the WGMOS detection model proposed in this paper with the introduction of the information entropy feature selection logic has better pixel-level defect detection performance than the traditional POL detection method, and the experiment fully verifies the robustness and effectiveness of this model.

Compared to manual inspection, this solution uses information entropy to automatically identify key pixels, eliminating 80% of redundant calculations. Results are quantified as confidence scores, putting an end to subjective manual inspections that merely conclude “it looks clean.” With 24/7 online monitoring, it eliminates the need for manual re-inspections and reduces rework costs.

This research proposal has numerous practical applications in the field of optical fiber manufacturing. After fiber drawing, each fiber must undergo end-face inspection. Traditional manual microscopic inspection is slow and prone to high rates of missed defects. While online automated solutions are faster, they often slow down production line throughput due to the high dimensionality of high-resolution image features. By using information entropy to perform “feature filtering” first, only a small number of indicators that best reveal scratches, dirt spots, and chipping are retained. This ensures that “automatically rejecting defective fibers before shipment” becomes a source of productivity rather than a bottleneck.

In practical application, this approach faces several issues that require further optimization. Since the entropy value considers only “information content” without distinguishing between “true defects” and “false defects,” it tends to treat textural noise as a high-value feature, leading to overfitting and failure to detect genuine scratches. A large number of pre-labeled end-face images must be prepared in advance; even minor dust or changes in lighting can cause abrupt shifts in the entropy ranking, and relabeling on-site to establish a baseline is time consuming.

## 5. Conclusions

This paper introduces the information entropy in information theory as the core criterion for defect feature screening and proposes the WGMOS detection method for multimode fiber end-face defect recognition. It is specifically designed for typical surface-type defects on the end face. By comparing the gray-scale differences of neighboring pixels, it can accurately distinguish high-entropy defect features from low-entropy backgrounds and interference noise and can be directly applied to actual production scenarios. Currently, there are few mature detection schemes for multimode fiber finished product end-face defects. The traditional POL detection method does not make targeted optimizations for the differences in the information entropy distribution between defects and backgrounds, and there is still room for improvement in detection accuracy and anti-interference ability. This paper also proposes an evaluation standard for multimode fiber end-face defects based on regional segmentation. The results of image demonstrations and quantitative data analysis verified the robustness and performance advantages of the WGMOS detection model: compared with the POL detection method, the equilibrium value and signal-to-noise ratio of multimode fiber end-face defect detection using the WGMOS detection model are increased by at least 38.20% and 6.0%, respectively. It can filter out more low-entropy redundant interference noise and achieve higher-precision defect detection.

## Figures and Tables

**Table 1:** Acceptance criteria for multimode fiber end faces under 200× microscope magnification.

Receiving and Inspection Area	Area Size	Foreign Matter or Stains That Cannot Be Wiped Off, Criteria for Determination	Scratch, Criteria for Determination
Area M1: Fiber optic cores	φ0 um ≤ A ≤ φ66 um	φ < 5 um, 5 or less passes Φ ≥ 5 um, Fail	widths < 3 um, 5 or fewer passes widths ≥ 3 um, Fail
Area M2: Fiber optic end face	φ66 um < B ≤ φ115 um	φ < 5 um, unrestricted 10 um > Φ ≥ 5 um, 8 or less passes Φ ≥ 10 um, Fail	widths < 3 um, 5 or fewer passes widths ≥ 3 um, Fail
Area M3: Fiber optic end face	φ115 um < C ≤ φ125 um	Not required	Not required
Area M4: Ceramic ends	φ125 um < D ≤ φ250 um	φ < 20 um, unrestricted Φ ≥ 20 um, Fail	Not required

**Table 2:** Equalization values and signal-to-noise ratio data for multimode fiber end faces under POL and WGMOS detection methods.

Serial No.	POL Equalized Value	WGMOS Equalized Value	Equalized Value Improvement	POL Signal-to-Noise Ratio	WGMOS Signal-to-Noise Ratio	Signal-to-Noise Ratio Improvement
1	1,285,544.69	2,329,230.45	81.19%	0.16	0.17	6%
2	1,531,082.98	2,197,185.12	43.51%	0.15	0.16	7%
3	1,400,447.99	2,223,329.66	58.76%	0.15	0.16	7%
4	3,036,005	4,195,779.52	38.20%	0.18	0.2	11%
5	4,310,192.35	9,402,518.97	118.15%	0.21	0.25	19%

## Data Availability

The original contributions presented in this study are included in the article. Further inquiries can be directed to the corresponding author.
